# Pediatric Hair Cycle Disorder With Persistent Short Hair Length

**DOI:** 10.7759/cureus.113958

**Published:** 2026-08-04

**Authors:** Kayla Coffman, Sean Hill, Shane Cook

**Affiliations:** 1 Department of Dermatology, Marshall University Joan C. Edwards School of Medicine, Huntington, USA

**Keywords:** loose anagen syndrome, pediatric alopecia, pediatric hair cycle disorder, psychological consequences, short anagen syndrome

## Abstract

Hair cycle disorders such as loose anagen syndrome (LAS) and short anagen syndrome (SAS) are rare conditions that can result in abnormal hair growth and an inability to grow hair beyond a certain length. LAS and SAS have significant clinical overlap despite distinct underlying pathophysiological mechanisms. This report describes the case of a five-year-old male who presented to dermatology with persistent short hair length and findings suggestive of a hair cycle disorder. Although the prognosis of both conditions is generally favorable, they may be associated with psychological impacts for patients and their families.

## Introduction

Pediatric alopecia can be divided into acquired and congenital conditions. Acquired conditions can be further subdivided into scarring alopecia, where hair follicles are permanently damaged, and non-scarring alopecia, where follicles are preserved. Congenital hair loss can be categorized as diffuse or focal. Hair loss disorders that affect pediatric patients encompass a broad range of categories, including alopecia areata, tinea capitis, traumatic hair loss, hair cycle disturbances, and congenital conditions. Hair cycle disturbances include telogen effluvium, anagen effluvium, loose anagen syndrome (LAS), and short anagen syndrome (SAS) [1].

SAS and LAS are rare conditions with limited cases in the literature. Although no true risk factors have been identified, these conditions most commonly affect young Caucasian girls with light-colored hair. Patients often present with limited hair growth that does not require frequent haircuts [2, 3]. SAS has no specific types, while LAS can be classified into three different groups based on hair reduction and other characteristics. LAS type A is sparse, short hair, while type B is diffuse or patchy, unruly hair. LAS type C most commonly affects adults with excessive shedding of loose anagen hairs [2].

The true prevalence and incidence of SAS are currently unknown, most likely due to rare occurrence and underrecognition [4]. LAS is uncommon, with an estimated incidence of approximately two cases per million per year and an average age of onset of three years. Parents of children with LAS may describe their child's hair as "matted" and "unmanageable" and may report diffuse hair loss. LAS patients typically do not have complete hair loss, but there is a classical association with occipital scalp thinning [5].

Hair growth occurs in three main phases, including anagen, catagen, and telogen. The anagen phase is known as the active growth phase, and 90% of hair follicles are in this state. The catagen phase is the transitional state and consists of around 2% of hair follicles. The telogen phase, also known as the dormant phase, comprises 5%-10% of hair follicles [1]. SAS is due to a shortened anagen phase in the hair cycle, causing premature transition of follicles into telogen and ultimately resulting in limited hair shaft length [6]. LAS is characterized by irregular anagen hair anchoring within the follicle, resulting in increased hair shedding [7].

The differential diagnosis for persistent short hair length in children includes hair cycle disorders, congenital or early-onset alopecia, acquired alopecia, and secondary causes of hair abnormalities. A thorough clinical evaluation, supplemented by diagnostic tests such as hair pull testing, trichoscopy, and trichogram, can help distinguish these conditions and guide appropriate management. Treatment is generally conservative and includes gentle hair care and topical minoxidil [4,5]. These disorders can negatively impact quality of life and contribute to psychological complications such as depression and anxiety [8].

We present a case of a five-year-old male with a history of poor hair growth and areas of bilateral temporal alopecia. This clinical presentation was suggestive of a pediatric hair cycle disorder characterized by an inability to grow hair beyond a certain length. This case highlights the diagnostic complexity and overlap of rare hair cycle disorders such as LAS and SAS.

## Case presentation

A five-year-old male presented to the dermatology clinic with a history of abnormal hair growth characterized by reduced length, areas of bilateral temporal alopecia, and fine hair. His past medical history was notable for prematurity with birth at 34 weeks 6 days gestation, requiring a four-day stay in the neonatal intensive care unit. Family history was significant for thyroid cancer, with no known history of hair disorders or alopecia. Immunizations have remained up to date with no history of severe illness. 

The patient’s hair was first noted at his four-year well-child visit when his parents expressed concern regarding his hairline and lack of hair growth. At that time, his pediatrician recommended a professional haircut and vitamin supplementation. At follow-up four months later, the patient had not received a haircut but had been adhering to vitamin supplementation with no improvement in hair growth. One year later, at his five-year well-child visit, poor hair growth remained an issue along with behavioral concerns. Growth parameters, including weight and body mass index, were within normal limits. Height was at the 5^th^ percentile but demonstrated appropriate tracking along the growth curve. Laboratory evaluation, including complete blood count, comprehensive metabolic panel, thyroid panel, and antinuclear antibody labs, was within normal limits. Key laboratory findings are summarized in Table 1. The patient was subsequently diagnosed with attention-deficit/hyperactivity disorder and was referred to dermatology for further evaluation of hair abnormalities and psychology for additional support. 

**Table 1:** Key laboratory findings Abbreviations: AST= aspartate aminotransferase; ALT= alanine aminotransferase; TSH= thyroid-stimulating hormone; T4= thyroxine; IFA= indirect immunofluorescence assay

Test	Result	Reference Range
White Blood Cell Count	5.2 k/cmm	4.5-13.5 k/cmm
Hemoglobin	12.6 gm/dL	11.5-13.5 gm/dL
Platelets	289 k/cmm	150-440 k/cmm
Glucose	75 mg/dL	74-109 mg/dL
Creatinine	0.48 mg/dL	0.44-0.65 mg/dL
AST	28 unit/L	15-50 unit/L
ALT	19 unit/L	7-52 unit/L
TSH	1.220 mIU/L	0.340-4.820 mIU/L
Free T4	0.96 ng/dL	0.76-1.46 ng/dL
Antinuclear Antibodies, IFA	Negative	Negative

**Figure 1:**
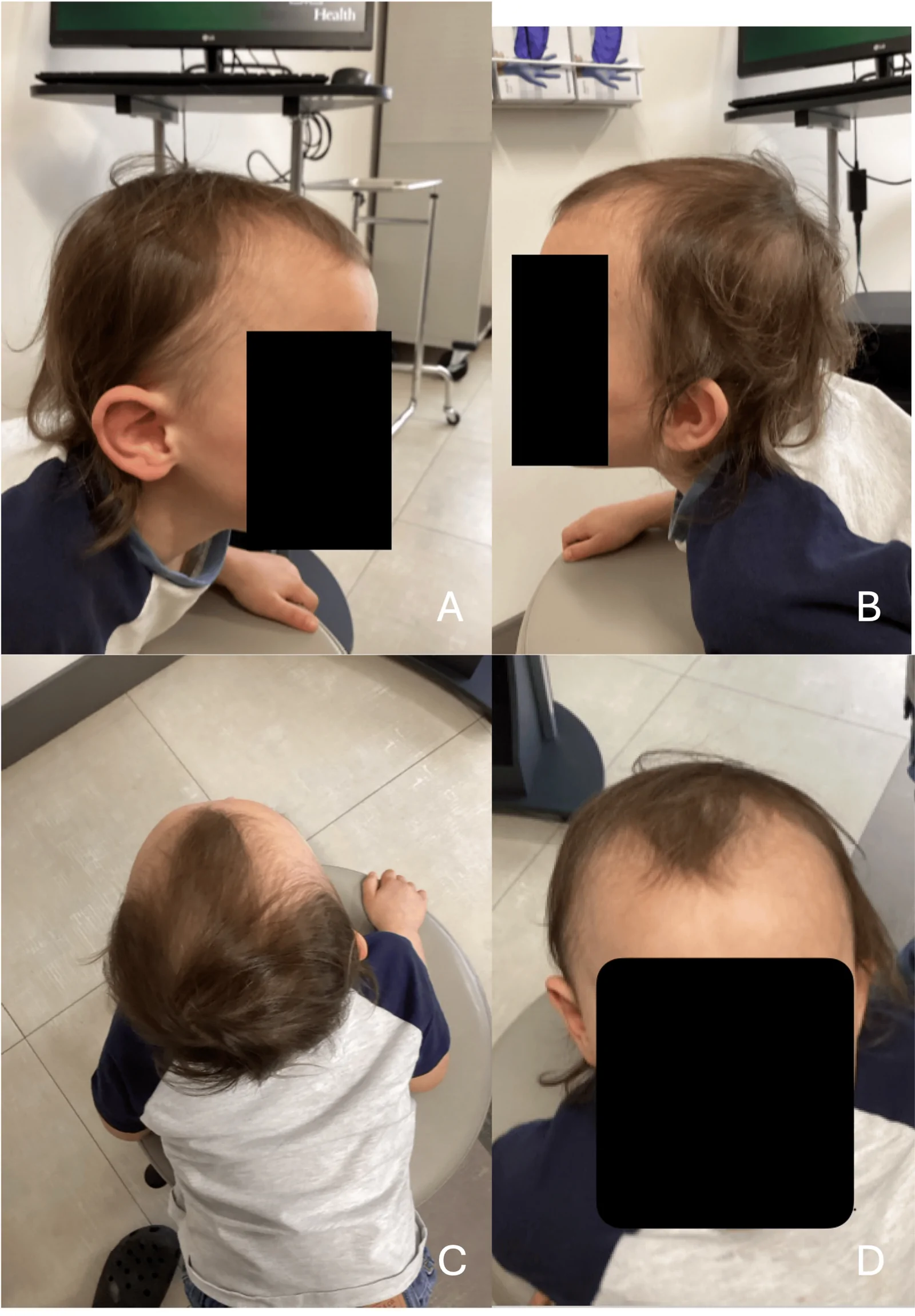
Clinical photographs of the scalp demonstrating short hair length. (A) right lateral view; (B) left lateral view; (C) vertex view; (D) frontal view.

Two weeks later, the patient presented to the dermatology clinic. Parents reported that, since birth, the patient's scalp hair had been markedly thin bilaterally and had never required haircuts. A physical exam demonstrated normal facial, eyelid, and ear findings, with bilateral temporal scalp alopecia, light-colored hair growth, and very fine, short hairs (Figure 1). The patient was referred to endocrinology for further evaluation of potential underlying metabolic etiologies. Referral to pediatric dermatology was recommended for specialized hair evaluation and diagnostic studies; however, parents elected to first pursue treatment with topical minoxidil prior to specialty evaluation. Through shared decision-making, scalp biopsy was deferred. Topical minoxidil 5% was prescribed twice daily to promote hair growth. Photographs were obtained to monitor treatment response, with plans for follow-up in three months. The patient did not attend any specialty appointments, including endocrinology, and was subsequently lost to follow-up.

## Discussion

The patient's presentation overlapped with several pediatric hair growth disorders, making diagnosis challenging. Hair pull testing, trichoscopy, and trichogram analysis can offer supportive evidence to distinguish these conditions. In patients with LAS, a hair pull test typically results in the painless extraction of more than 10 loose anagen hairs, compared with only one or two hairs in a healthy individual [7]. However, the test may be negative if loose hairs have already been removed from routine brushing and styling. Therefore, obtaining a history of patients' hair care may assist in interpreting the results [3]. On trichogram analysis, the plucked anagen hair characteristically has a “floppy sock” appearance, with a twisted root and ruffling of the cuticle [7]. In contrast, patients with SAS may also have a positive hair pull test but can be distinguished by the characteristic short telogen hairs with a tipped point on trichogram analysis [6]. 

A broad differential diagnosis for persistent short hair length in children must be considered and include hair cycle disorders, congenital or early-onset alopecia, acquired alopecia, and secondary causes of hair abnormalities. Congenital alopecia areata can present in children with patchy hair loss that can progress to total hair loss of the scalp or body, depending on the severity [9]. Temporal triangular alopecia is a non-scarring form of alopecia that typically appears around birth or before a patient turns 10 years old. It presents as an alopecic patch or patches along the frontotemporal region, rarely the occipital region, that will persist throughout life if not treated [10]. Acquired conditions such as trichotillomania and telogen effluvium can be triggered in childhood or adolescence by traumatic or stressful experiences. Secondary causes of alopecia include conditions affecting the hormonal regulation of the hair cycle. Thyroid dysfunction, androgen excess associated with polycystic ovarian syndrome or congenital adrenal hyperplasia, and cortisol excess in Cushing syndrome must be considered and ruled out [11].

Pediatric patients with LAS have an overall good prognosis, and most will have spontaneous recovery by adulthood. First-line treatment is topical minoxidil due to its proposed mechanism of prolongation of the anagen phase. Gentle hair care is also recommended to avoid external stress on the hair. Biotin has not been shown to be beneficial to patients with LAS [5]. In patients with SAS, the prognosis is also favorable, and a slow, spontaneous improvement in hair growth is reported with age. A retrospective review of 25 patients with SAS revealed that biotin in combination with minoxidil and biotin alone are effective treatment options [4]. 

The psychological impact on patients with rare conditions such as LAS and SAS is an important consideration for clinicians. A study published in 2022 retrieved data from a non-validated online questionnaire from members of a Facebook support group for SAS and LAS. Members self-reported that they had been diagnosed with LAS or SAS by a dermatologist and answered the questionnaire regarding the psychological consequences of their disease. Of 163 participants, 89 had LAS and 74 had SAS [8].

Among patients with LAS, the mean age of symptom onset was 2.3 years, and the mean age of diagnosis was 5.3 years. The cohort was predominantly female (98.9%) and white (95.5%). Approximately one-third (33.7%) reported bullying or mistreatment from peers. Negative psychological symptoms were reported in 42.7%, while 48.3% of their caregivers suffered psychological distress. The number of patients receiving psychological treatment was 2.6% [8]. In comparison, patients with SAS were also predominantly White female patients with a similar age of symptom onset and diagnosis. However, patients with SAS reported a higher percentage of negative psychological consequences (56.7%) and a higher percentage of patients receiving psychological treatment (9.5%) [8]. 

Although larger standardized studies need to be conducted, this study highlights the psychological burden associated with two rare pediatric disorders. Providers must recognize that even though these two conditions may remit in the future, it does not diminish the impact of the disease in its active state. Clinicians should discuss and provide necessary mental health resources to patients and their families [8]. 

A limitation of this case is the absence of hair pull testing, trichoscopy, and trichogram. These minimally invasive techniques provide supportive findings that may aid in differentiating LAS and SAS. Although scalp biopsy has been described as a diagnostic tool and may be considered in select cases, it was deferred in this case pending further specialty evaluation. Referral to pediatric dermatology was recommended given their specialized expertise in pediatric hair disorders and interpretation of specialized hair studies. The family elected to first pursue topical treatment with scheduled follow-up in three months. Unfortunately, the patient was lost to follow-up, limiting the ability to obtain additional diagnostic evaluation and distinguish between LAS and SAS.

## Conclusions

This case highlights the limited number of reported pediatric hair cycle disorders and underscores the importance of increasing clinician awareness. Greater recognition and reporting of these conditions may improve our understanding of their epidemiology, demographic patterns, potential risk factors, phenotypic variations, and clinical course, ultimately contributing to earlier diagnosis and more informed management. Physicians can face challenges in diagnosing pediatric hair cycle disorders, and a broad differential diagnosis is often needed. Given the poor hair growth, abnormal hair length, and bilateral temporal alopecia in our patient, LAS and SAS were the top differential diagnoses.

A thorough history, physical examination, and evaluation for underlying metabolic, endocrine, nutritional, and genetic disorders are essential when assessing pediatric hair conditions. Early recognition and appropriate referral can help avoid unnecessary interventions, guide management, and address the psychosocial impact of hair disorders on affected children and their families. A multidisciplinary approach to the evaluation and management of uncommon pediatric hair cycle disorders allows for comprehensive patient support and care.
